# Empowering women: intimate partner violence and its association with unintended pregnancies, contraceptive use, and HIV infection among Ugandan women: a cross-sectional population-based study in Wakiso and Hoima districts

**DOI:** 10.1080/16549716.2025.2585674

**Published:** 2025-12-01

**Authors:** Elisabet Beseran Valero, Emmanuel Kyasanku, Robert Bulamba, Philip Kato, Erinah Nabunya, Fred Nalugoda, Anna Mia Ekström, Malachi Ochieng Arunda

**Affiliations:** a Department of Global Public Health, Karolinska Institutet, Stockholm, Sweden; b Africa Medical and Behavioural Sciences Organization (AMBSO), Hoima, Uganda; c Department of Infectious Diseases/Venhälsan, South General Hospital, Stockholm, Sweden

**Keywords:** gender based violence, sexual and reproductive health, Uganda, sexual and reproductive rights, reproductive health outcomes

## Abstract

**Background:**

While the adverse effects of intimate partner violence (IPV) on sexual and reproductive health are globally recognized, research in low- and middle-income countries, particularly Uganda, remains limited.

**Objective:**

This study aims to assess the association between past-year IPV and HIV, contraceptive use, and unintended pregnancies among Ugandan women.

**Methods:**

Data from the Africa Medical and Behavioural Sciences Organization collected during the 2019 Population Health Surveillance in Hoima and Wakiso districts, Uganda were analysed including 1,819 women aged 15–49. Binary logistic regression was performed to estimate adjusted odds ratios (AOR) at 95% confidence interval. This study used a cross-sectional design; therefore, causal inference cannot be established.

**Results:**

Women with past-year sexual IPV had 3.2 times higher odds of having unintended pregnancy (adjusted odds ratio (AOR): 3.2, 95% CI: 1.72, 5.97). There was a borderline association between both HIV infection and physical (AOR: 1.67 95% CI: 1.00, 2.79) and psychological IPV (AOR: 1.42 95% CI: 1.00, 2.00). Contraceptive use was not found to be associated with IPV (AOR: 1.30 95% CI: 0.90, 1.89).

**Conclusion:**

The results indicate that the link between IPV and unintended pregnancies is suggestive of a potential association. While the association between IPV and HIV was not statistically significant, it points to a possible relationship that requires further research. There was no significant association between IPV and contraceptive use. Given the design of the study, causal inference cannot be established; however, the results may inform future studies aimed at preventing IPV and improving SRHR outcomes in Uganda.

## PAPER CONTEXT

- **Main findings:** Intimate partner violence is linked to unintended pregnancies, and although not significant, this violence might be associated to HIV infection among women of reproductive age in Wakiso (urban) and Hoima (semiurban) districts in Uganda.
- **Added knowledge:** This result adds to the emerging body of knowledge that links intimate partner violence to poor sexual and reproductive health outcomes among women in low-resource settings.
- **Global health impact for policy and action:** Policies designed to prevent intimate partner violence should address its connections with unintended pregnancies and HIV infection within this population to ensure comprehensive public health interventions.

## Background

Intimate partner violence (IPV) is a worldwide public health problem that impacts women’s mental, physical, and reproductive health [1]. The World Health Organization (WHO) estimated that 15–71% of women experience IPV in their lifetime [2]. IPV refers to physical violence, sexual violence, or psychological aggression by a current or former intimate partner [1]. IPV not only causes harm but also affects sexual and reproductive health and rights (SRHR), which is ‘a state of physical, emotional, mental, and social wellbeing in relation to all aspects of sexuality and reproduction, not merely the absence of disease, dysfunction, or infirmity’ [3]. SRHR includes the right to make personal choices regarding one’s body and access services that support these rights, as they are part of human rights [3].

The theory of gender and power provides a framework to understand the link between IPV and SRHR. It identifies three interrelated structures in male–female relationships that contribute to IPV perpetration: the sexual division of labour, the sexual division of power, and the structure of emotional attachments [4]. For instance, women often handling unpaid work, such as child care, creates economic dependence on their male partners, reinforcing power imbalances and strengthening the emotional attachment [5]. Social norms further support traditional gender roles [5], such as the expectation that men control sexual relationships, which may pressure women to stay in challenging situations [6]. These dynamics contribute to IPV perpetration by male partners and to adverse SRHR outcomes among women [7]. Guided by this theory, we hypothesised that women experiencing IPV would be less likely to use contraception and more likely to report HIV infection or unintended pregnancy.

### Linking IPV and sexual and reproductive health and rights

Several pathways explain how IPV affects SRHR. IPV, rooted in gendered power imbalances, uses fear and control to reduce women’s autonomy, limiting their ability to consent to sex or protect against unintended pregnancies, HIV, and other Sexually Transmitted Infections (STIs) [8–10]. It also restricts women’s access to reproductive health services like contraception [10,11]. This hinders care-seeking, making it harder to access treatment and screening. IPV may lead to reproductive coercion, including pregnancy pressure and contraception sabotage. Additionally, physical or sexual violence can directly result in unintended pregnancies and increased HIV/STI risk by preventing contraception use [12].

Several studies examined the association between IPV and SRHR outcomes [13–22]. IPV has been linked to increased vulnerability to HIV among women [13–15,23,24]. For example, a South African cohort study revealed a higher HIV incidence rate in women who experienced sexual or physical IPV [13]. This association could be explained by power imbalances, male control, and coercion into high HIV-risk behaviours, such as forced unprotected sex and substance use [16,17].

Intimate Partner Violence has also been associated with unintended pregnancies. Studies in sub-Saharan Africa and the US found higher odds of unintended pregnancies among women experiencing sexual violence, with reproductive coercion and birth control sabotage as key risk factors [9,19].

There are discrepancies in research on contraceptive use and IPV. Some studies report higher contraception use among IPV survivors [20,25], possibly to prevent pregnancy in a conflictual relationship, while others found reduced use of contraception [21] potentially to prevent more violence from happening. This different context in which IPV occurs might be a potential explanation for these differences [22]. These divergent findings highlight that IPV influences contraceptive behaviors in complex, context-specific ways – depending on whether women are able to exercise agency or are constrained by coercive dynamics.

The link between IPV, HIV, unintended pregnancies, and contraceptive use, is especially critical in low- and middle-income settings. In sub-Saharan Africa, girls and young women aged 15–24 years accounted for over 77% of new HIV infections in 2022 [26]. Low contraceptive use also adds to the problem; in Uganda in 2019, only 9.4% of adolescents girls used modern contraceptives [27]. Uganda also has high unintended pregnancy rates, with 145 per 1000 women affected [28], and adolescents aged 15–19 years at the highest risk [29]. Additionally, 55% of ever-married women in Uganda experienced IPV in 2016 [30].

Empirical research on the links between IPV and SRHR outcomes – HIV, contraceptive use, and unintended pregnancies in Uganda – is limited. Two studies found higher HIV rates among IPV survivors, though only one included women aged 15–24 and excluded psychological violence [31]. Findings on IPV and contraceptive use show conflicting results: one study found IPV survivors were less likely to use partner-dependent contraception but more likely to use hidden methods, while others found no associations [32]. Studies on unintended pregnancies suggest a higher risk for women experiencing sexual IPV [18,33] but have not elucidated the role of physical or psychological violence. Therefore, this research addresses the knowledge gap on how different types of IPV are associated with key SRHR outcomes; unintended pregnancy, contraception use, and HIV, among women of reproductive age in Uganda, as understanding these differentiated associations is crucial to inform targeted interventions in this context.

## Methods

### Study design, data collection, and study population

This study utilizes secondary data from a cross-sectional survey conducted in Wakiso and Hoima districts in Uganda. The study is based on data collected by the Africa Medical and Behavioural Sciences Organization (AMBSO) through the Population Health Surveillance (APHS) in 2019. Established in 2018, the APHS is an open, longitudinal, population-based cohort that includes individuals aged 13 and above [34]. Data was collected from Wakiso district in central Uganda and Hoima district in western region, encompassing urban, rural, and semi-urban communities. These areas were chosen to capture a variety of community types, urbanization levels, and diverse socioeconomic status. Africa Medical and Behavioural Sciences Organization conducts the survey annually, gathering information on reproductive health, sociodemographic, mental health, experience of IPV, and communicable and non-communicable diseases [34].

This study uses data from APHS2 the fourth round follow-up survey of the Population Health Surveillance conducted by AMBSO in 2019. A total of 2,363 women aged 15–49 were expected to answer the survey. The response rate was 77%.

This study uses a cross-sectional design; therefore, the aim is to identify potential associations, rather than causality, as temporality cannot be determined.

### Data analysis

The three outcome variables for this study were HIV status, current contraceptive use, and unintended pregnancies, which were dichotomized as follows. *HIV*: Participants were asked the result of the most recent HIV test, with responses categorized as, (1 = Yes, 0 = No). *Current contraception use*: Participants were asked which family planning methods they were currently using, including *modern* (e.g. pills, condom, and IUDs) and *traditional methods* (Herbs/traditional medicine, breastfeeding, and withdrawal). This variable was dichotomized into modern family planning use (1) and non-use or traditional method use (0). *Unintended pregnancy*: Participants were asked how many of the pregnancies were unintended. This variable was dichotomized into those had at least one unintended pregnancy (1 = Yes) and those who never had an unintended pregnancy (0 = No).

The exposure variable was the experience of IPV in the past 12 months (past year). Questions about sexual and physical violence were adapted for the Ugandan context by AMBSO from a World Health Organization-validated standard tool [35]. Psychological violence was not measured using the full standard module. Nevertheless, our study incorporated two questions that are closely related to standard psychological IPV items and reflect core aspects of the construct as outlined in widely used instruments, including the WHO Violence Against Women Instrument and the DHS Domestic Violence Module.

The three exposure variables for this study were past 12 months physical, sexual, and psychological IPV, which were dichotomized as follows.

*Physical IPV*: Participants were asked whether in the past 12 months, they have been pushed, slapped or held down, punched with a fist or with something that could hurt, kicked or dragged, strangled or burned, threatened with a knife, gun, or other weapon, or have been attacked with a knife, gun, or other type of weapon by their sexual partner. Responses were dichotomized into `yes´ (1) and `no´ (0).

*Sexual IPV*: Participants were asked if they have been threatened to have sex against their will, physically forced to have sex, or forced to perform sexual acts that the participant did not want to by their partner in past 12 months. Responses were dichotomized into `yes´ (1) and `no´ (0).

*Psychological IPV*: Participants were asked if they have been being shouted, or verbally abused by their sexual partner in the past 12 months. Responses were also dichotomized into `yes´ (1) and `no´ (0).

*Sociodemographic, sex and alcohol-related variables* collected included age of the respondent, categorized into 15–24, 25–34, 35–44, 45–49 years, age at first sex (≤14,15–19, 20–30), age at first marriage (≤14,15–19, 20–42), place of residence (1 = Semi-urban 2 = Rural 3 = Urban), education (1 = No education/Primary education which included lower primary, upper primary, primary apprenticeship and O’ Level apprenticeship 2 = Secondary education which included lower secondary and higher secondary 3 = Higher education which included Technical/University, Primary professional and O-Level professional), currently married (1 = Yes 2 = No), religion (1 = Catholic, 2 = Protestant, 3 = Other Christian, 4 = Muslim, 5 = Other), occupation (1 = Formal employment, 2 = Farmers, 3 = Self-employed, 4 = Students) and alcohol consumption (0 = No, 1 = Yes).

Binary logistic regression was used to analyze the association between past-year sexual, physical, psychological, and any IPV with the SRHR outcomes, as both the outcome and exposure variables were dichotomous. Crude (Table 3) and adjusted (Table 4) analyses were performed excluding missing data and responses marked ‘Not applicable’ or ‘No partner’.

The selection of the confounders was based on a review of previous literature and theoretical knowledge in the field. The confounders selected included education level, age, marital status, place of residence, age at first sex, occupation, and alcohol use which were reported in the literature to be associated with IPV [36] and also with HIV, unintended pregnancies, and contraceptive use in Uganda [37].

## Results

### Population characteristics

The study included 1,819 women aged 15–49 from Hoima and Wakiso districts in Uganda who completed the 2019 APHS follow-up survey. Population characteristics are presented in Tables 1 and 2. Most participants (73%) were aged 15–34, with varied educational levels, predominantly no formal education, primary or secondary education. Most women lived in urban setting, were married, and self-employed.

**Table 1.** Distribution of sociodemographic, sex-, and alcohol-related variables by past-year experience of IPV among women aged 15–49 in Hoima and Wakiso, 2019.

Characteristics	Past 12 months IPV (Sexual, physical, psychological)				
	N = 1,458				
No		Yes	p-value		
	n	%	n	%	
**Education level**					
No or primary	316	46.7	393	50.3	0.39
Secondary	298	44.1	317	40.5	
Higher	62	9.2	72	9.2	
**Age (years)**					
15–24	220	32.5	231	31.4	0.62
25–34	278	41.1	340	43.5	
35–44	137	20.3	166	21.2	
45–49	41	6.1	45	5.7	
**In current marriage**					
Yes	435	64.3	577	73.9	0.001
No	241	35.6	204	26.1	
**Age at first marriage**					
≤14	26	4.7	40	5.8	0.11
15–19	269	48.6	367	53.3	
20–42	259	46.7	282	40.9	
**Place of residence**					
Semi-urban	240	35.5	219	28.0	0.003
Rural	175	25.9	254	32.5	
Urban	261	38.6	309	39.5	
**Age at first sex**					
≤14	92	14.0	134	17.5	0.20
15–19	480	73.3	537	70.3	
20–30	83	12.7	93	12.2	
**Occupation**					
Formal employment	97	14.3	76	9.7	0.001
Farmers	122	18.0	260	33.2	
Self-employed	433	64.0	434	55.5	
Students	24	3.5	12	1.5	
**Alcohol use**					0.004
Yes	165	24.4	244	31.2	
No	511	75.6	538	68.8	
**Average**	676	46.36	782	53.64	

*P*-values generated from chi-square analysis, statistical significance (*p* < 0.05 two-sided).

IPV – Intimate partner Violence.

**Table 2.** Sociodemographic, sex-, and alcohol-related characteristics by SRHR outcomes among women aged 15–49 in Hoima and Wakiso, 2019.

Characteristics	HIV infection					Unintended pregnancies					Contraceptive use				
	N = 1,817					N = 1,651					N = 975				
	Negative		Positive		p-value	No		Yes	%	p-value	No/Traditional		Modern		p-value
	n	%	n	%		n	%	n	%		n	%	n	%	
**Education level**															
No or Primary	683	42.7	162	74.0	0.001	404	50.2	407	48.1	0.003	70	37.6	372	47.1	0.03
Secondary	756	47.3	52	23.7		319	39.6	386	45.6		89	47.8	340	43.0	
Higher	159	9.9	5	2.3		82	10.2	53	6.3		27	14.5	77	9.8	
**Age (years)**															
15–24	634	39.7	33	16.2	0.001	199	24.7	328	38.8	0.001	68	36.6	258	32.70	0.07
25–34	581	36.4	80	36.5		368	45.7	268	31.7		65	35.0	357	45.2	
35–44	284	17.8	81	37.0		184	22.9	180	21.3		43	23.1	140	17.7	
45–49	99	6.2	25	11.4		54	6.7	70	8.3		10	5.4	34	4.3	
**Current marriage**															
Yes	912	57.1	118	53.9	0.36	567	70.4	443	52.4	0.001	113	60.7	528	66.9	0.11
No	685	42.9	101	46.1		238	29.6	403	47.6		73	39.2	261	33.1	
**Age at first marriage (years)**															
≤14	49	4.2	20	9.8	0.003	26	3.5	42	7.0	0.001	5	3.6	35	5.3	0.09
15–19	595	51.1	103	50.2		368	49.2	317	53.0		60	43.8	347	52.2	
20–42	520	44.7	82	40.0		354	47.3	240	40.0		72	52.5	283	42.6	
**Place of residence**															
Semi-urban	463	29.0	80	36.5	0.01	248	30.8	263	31.1	0.001	54	29.0	261	33.1	0.26
Rural	462	28.9	71	32.4		271	33.7	213	25.2		49	26.3	227	28.8	
Urban	673	42.1	68	31.0		286	35.5	370	43.7		83	44.6	301	38.1	
**Age at first sex (years)**															
≤14	197	14.2	52	24.4	0.001	75	9.6	150	23.0	0.001	24	13.3	118	15.3	0.008
15–19	995	71.8	146	68.5		572	73.2	449	68.8		119	66.1	564	73.0	
20–30	194	14.0	15	7.0		134	17.2	54	8.3		37	20.6	91	11.8	
**Occupation**															
Formal employment	191	11.9	18	8.2	0.001	97	12.0	86	10.2	0.001	24	12.9	98	12.4	0.001
Farmers	364	22.8	71	32.4		245	30.4	176	20.8		30	16.1	227	28.8	
Self-employed	870	54.4	129	58.9		462	57.4	457	54.0		114	61.3	447	56.6	
Students	173	10.8	1	0.5		1	0.1	127	15.0		18	9.7	17	2.1	
**Alcohol use**					0.001					0.536					0.01
Yes	368	23.0	141	64.4		194	24.1	215	25.4		35	18.8	221	28.01	
No	1230	77.0	78	35.6		611	75.9	631	74.6		151	81.2	568	72.0	
**Average**	1,580	87.9	219	12.0		805	48.9	846	51.2		186	19.1	789	80.9	

*P*-values generated from chi-square analysis, statistical significance (*p* < 0.05 two-sided).

SRHR – Sexual Reproductive Health and Rights.

Regarding sexual behavior, most women had their first sexual experience between the ages of 15–19, and nearly 60% married within that age range. Additionally, most of them reported not drinking alcohol.

### Univariate analysis of exposure and outcome

**Figure 1.**
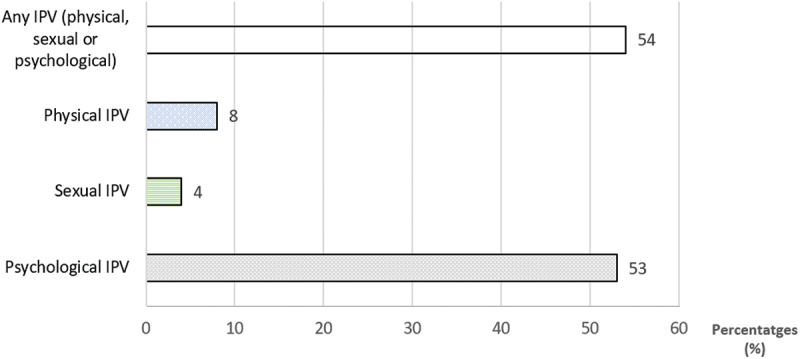
Prevalence of past 12 months IPV form among Ugandan women in Wakiso and Hoima, 2019. IPV – Intimate Partner Violence. Physical, sexual, and psychological IPV refer, respectively, to experiencing physical violence, sexual abuse or threats, and verbal abuse by a sexual partner in the past 12 months.

Table 1 presents the characteristics of the sample stratified by past 12 months IPV exposure and the results of the chi-square test that shows the association between IPV, demographic, sexual, and alcohol-related characteristics. About 50% respondents experienced IPV in the past year, with psychological IPV being the most prevalent (Figure 1). IPV exposure was significantly associated with marriage, place of residence, occupation, and alcohol use (*p* < 0.05)

**Figure 2.**
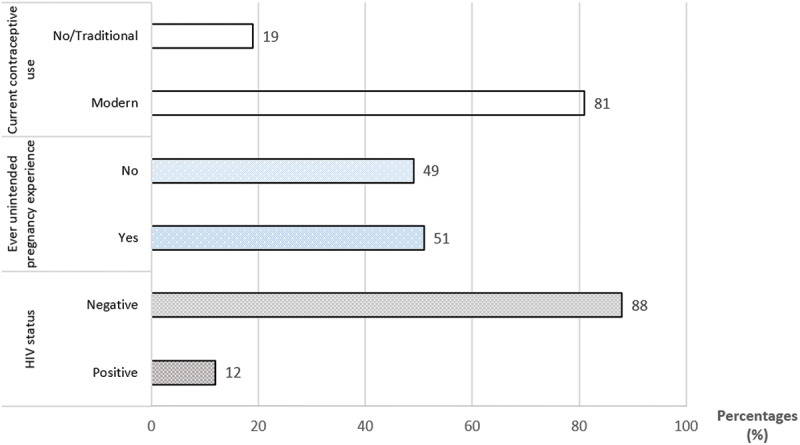
Prevalence of sexual and reproductive health and rights outcomes among Ugandan women in Wakiso and Hoima districts, 2019. Current contraceptive use includes: modern methods (condoms, pills, IUDs); non-use includes no contraception or traditional methods (herbs, breastfeeding, withdrawal). Ever unintended pregnancy: at least one unintended pregnancy. HIV status: result of the most recent test (positive or negative).

Table 2 shows the sample characteristics by sexual and reproductive health outcomes, with chi-square test results highlighting associations between outcomes and demographic, sex and alcohol-related factors. In the sample, 12% of the women were HIV positive, 51% had experienced an unintended pregnancy, and 80% were currently using modern contraceptive (Figure 2).

Education, age, age at first sex, and occupation were associated with all three outcomes (*p* < 0.05). Age at first marriage and place of residence were linked to HIV and unintended pregnancies, while alcohol use was associated with HIV and contraceptive use.

### Logistic regression analysis

Table 3 presents the unadjusted odds ratios (OR) from binary logistic regression examining the association between sexual, physical, and psychological IPV and the outcomes; HIV, contraceptive use, and unintended pregnancies. Physical and psychological IPV were linked to higher odds of HIV (physical IPV unadjusted OR [UOR] = 2.00 (CI: 1.26, 3.18)); psychological IPV, UOR = 1.42 (CI: 1.04, 1.95) (*N* = 1450). Women experiencing past-year sexual IPV had three times higher odds of unintended pregnancy (sexual IPV unadjusted OR [UOR] = 3.59 (CI: 1.98, 6.52)), while psychological IPV increased unadjusted OR by 1.58 (CI: 1.08,2.31) (*N* = 1325). No significant association was found between any type of past 12 months IPV and modern contraceptive use (*N* = 925).

**Table 3.** Unadjusted odds ratio for the associations between past-year experience of IPV, sociodemographic characteristics, and SRHR outcomes (HIV, contraception use, and unintended pregnancy) among women aged 15–49 in Hoima and Wakiso districts.

Characteristics	Categories	HIV positivity uOR (CI) *N* = 1450	Unintended pregnanciesuOR (CI) N = 1325	Contraceptive use uOR (CI) N = 925
**Past 12 months IPV**				
Any	No	1.00	1.00	1.00
	Yes	1.38 (1.00,1.89)	0.96 (0.77, 1.19)	1.40 (0.99, 1.98)
Physical	No	1.00	1.00	1.00
	Yes	2.00 (1.26, 3.18)	1.58 (1.08,2.31)	2.03 (0.91,4.51)
Psychological	No	1.00	1.00	1.00
	Yes	1.42 (1.04,1.95)	0.94 (0.76,1.17)	1.39 (0.98,1.96)
Sexual	No	1.00	1.00	1.00
	Yes	1.31 (0.66, 2.63)	3.59 (1.98,6.52)	0.73 (0.34,1.56)
**Education level**	No or primary	1.00	1.00	1.00
	Secondary	0.30 (0.21, 0.40)	1.20 (0.98, 1.47)	0.72 (0.51, 1.0)
	Higher	0.13 (0.05, 0.33)	0.64 (0.44,0.93)	0.54 (0.32,0.89)
**Age**	15–24	1.00	1.00	1.00
	25–34	2.64 (1.74, 4.03)	0.44 (0.35, 0.56)	1.45 (0.99, 2.11)
	35–44	5.48 (3.57, 8.41)	0.59 (0.45, 0.78)	0.86 (0.56, 1.32)
	45–49	4.85 (2.77,8.50)	0.78 (0.53, 1.17)	0.90 (0.42, 1.909)
**Marital status**	Married	1.00	1.00	1.00
	Not married	1.14 (0.86, 1.51)	2.17 (1.77,2.65)	0.76 (0.55, 1.06)
**Age at first marriage**	≤14	1.00	1.00	1.00
	15–19	0.42 (0.24, 0.74)	0.53 (0.32, 0.89)	0.83 (0.31, 2.19)
	20–42	0.39 (0.22, 0.68)	0.42 (0.25, 0.70)	0.56 (0.21,1.48)
**Place of residence**	Semi-urban	1.00	1.00	1.00
	Rural	0.89 (0.63,1.25)	0.74(0.57,0.95)	0.96, (0.63,1.47)
	Urban	0.58 (0.41,0.82)	1.22 (0.97,1.54)	0.75 (0.51, 1.10)
**Age at first sex**	≤14	1.00	1.00	1.00
	15–19	0.56 (0.39, 0.79)	0.39 (0.29, 0.53)	0.96 (0.60,1.56)
	20–30	0.29 (0.16, 0.54)	0.20 (0.13, 0.31)	0.50 (0.28, 0.89)
**Occupation**	Formal employment	1.00	1.00	1.00
	Farmers	2.07 (1.20, 3.57)	0.81 (0.57, 1.15)	1.85 (1.03, 3.33)
	Self-employed	1.57 (0.94, 2.64)	1.12 (0.81, 1.53)	0.96 (0.59,1.57)
	Student	0.06 (0.01, 0.46)	143.24 (19.60,1046.81)	0.23 (0.10, 0.51)
**Alcohol consumption**	No	1.00	1.00	1.00
	Yes	1.85 (1.37, 2.50)	1.07 (0.86, 1.34)	1.68 (1.13, 2.50)

uOR – Unadjusted Odds Ratio, IPV – Intimate Partner Violence, SRHR – Sexual Reproductive Health and Rights, CI – Confidence Intervals.

**Figure 3.**
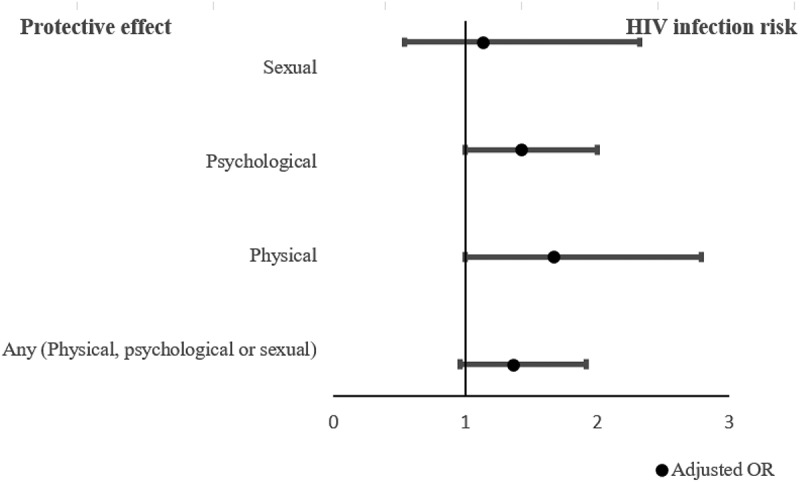
Forest plot illustrating adjusted odds ratio (AOR) and 95% confidence intervals for the associations between past-year experience of intimate partner violence and HIV among women aged 15–49 in Wakiso and Hoima, Uganda.

Table 4 presents adjusted ORs after controlling for covariates. The association between physical and psychological IPV with HIV was borderline significant, suggesting a potential association between those variables. (Physical IPV adjusted OR [AOR] = 1.67 (CI: 1.00, 2.79), psychological IPV AOR = 1.42 (CI: 1.00, 2.00) (*N* = 1380) (Figure 3).

**Table 4.** Adjusted odds ratio for experience of past-year IPV and SRHR outcomes among women aged 15–49 in Wakiso and Hoima districts, 2019.

Variable	Categories	HIV positivity AOR (CI)	Unintended pregnancies AOR (CI)	Contraceptive use AOR (CI)
		*N* = 1,380	*N* = 1,286	*N* = 903
**Past 12 months IPV**				
Any (physical, psychological and sexual)	No	1.00	1.00	1.00
	Yes	1.36 (0.96,1.92)	0.97 (0.76, 1.22)	1.30 (0.90, 1.89)
Physical	No	1.00	1.00	1.00
	Yes	1.67 (1.00, 2.79)	1.38 (0.92, 2.07)	1.81 (0.80, 4.10)
Psychological	No	1.00	1.00	1.00
	Yes	1.42 (1.00, 2.00)	0.94 (0.74, 1.19)	1.30 (0.89, 1.88)
Sexual	No	1.00	1.00	1.00
	Yes	1.13 (0.54, 2.32)	3.21 (1.72, 5.97)	0.69 (0.31, 1.52)

Adjusting for education, age, marital status, age at first sex, occupation, place of residence and alcohol use.

IPV – Intimate Partner Violence, SRHR – Sexual Reproductive Health and Rights, AOR – Adjusted Odds Ratio, CI – Confidence Intervals.

N indicate the sample size included in each analysis. Sample sizes were the same across different IPV types (Physical, sexual, and psychological).

**Figure 4.**
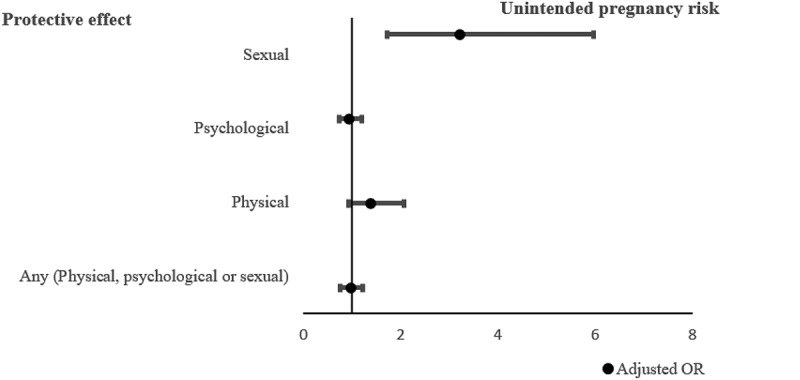
Forest plot illustrating adjusted adjusted odds ratio (AOR) and 95% confidence intervals for the associations between past-year experience of intimate partner violence and unintended pregnancies among women aged 15–49 in Wakiso and Hoima, Uganda.

Past-year sexual IPV was still strongly associated with unintended pregnancy AOR = 3.21 (CI: 1.72, 5.97). The result for physical IPV and unintended pregnancy was not significant, AOR = 1.38 (CI: 0.92, 2.07) *N* = 1286 (Figure 4).

After controlling for confounding, no significant association was found between any type of past 12 months IPV and contraceptive use, AOR = 1.30 (CI: 0.90, 1.89), *N* = 903.

## Discussion

The study aimed to explore the association between past 12 months IPV (physical, psychological, and sexual) and the following key SRHR outcomes; contraceptive use, unintended pregnancies, and HIV.

In examining the potential association between past-year IPV and HIV, the results did not reach statistical significance; nevertheless, the borderline significance observed may indicate a trend toward increased odds of HIV positivity among women experiencing physical or psychological IPV. This trend is consistent with findings from previous studies [13,14,23,24]. A larger sample size would likely be needed to detect this association in this study. The idea that psychological and physical violence limits women’s autonomy in condom use and increases their HIV risk could explain this potential association [13]. Additionally, men who perpetrate psychological IPV may engage in sexual risk behaviors such as multiple partners and substance use, further raising HIV risk [14].

This study did not find an association between sexual IPV and HIV, unlike previous research [23,24]. A possible reason for this inconsistency may be underreporting due to social desirability and recall bias, as sexual violence is often underdisclosed due to stigma and social norms [38], despite efforts to ensure privacy and confidentiality during data collection.

The results showed that women who experienced sexual IPV in the past year had significantly higher odds of unintended pregnancy, three times higher than those without experience of sexual IPV. This finding is consistent with other studies in sub-Saharan Africa [18]. Sexual violence may result in forced sexual intercourse, where contraception is not used, while power imbalances, such as reproductive coercion, can also play a role [9]. Social norms in Uganda, such as beliefs about women’s sexual availability and justification that there are instances where a woman can be beaten, may further perpetuate IPV and its reproductive health risks [39]. Since IPV is often recurrent, past-year violence likely indicates a history of abuse. However, given the cross-sectional design of this study, which cannot establish temporality, women with unintended pregnancy may also be vulnerable to recent sexual IPV. Therefore, it is not possible to determine whether unintended pregnancy preceded IPV or vice versa. In addition, this study did not find an association between physical or psychological IPV and unintended pregnancy, underscoring the need for further research into the complex mechanisms underlying these relationships.

The findings found no significant association between contraceptive use and IPV, consistent with previous research in Uganda [40], but contrasting with studies reporting either lower [32] or increased contraceptive use among women experiencing IPV in sub-Saharan Africa [20,25]. These disparities may stem from cultural differences in attitude toward violence and contraception. The high prevalence of modern contraceptive use in this sample (80%) is substantially higher than the national average in the 2022 Uganda Demographic Health Survey (33%) [41]. While urban bias is a common explanation for such discrepancies [42], our study design minimised this risk by including participants from urban, peri-urban, and rural areas in Wakiso and Hoima districts. Other factors that are likely to have contributed are the non-response among younger participants – who typically report lower contraceptive use nationally – and may have disproportionately excluded non-users, thereby inflating prevalence. Social desirability bias cannot be ruled out, as participants may have been more willing to report contraceptive use while underreporting sensitive experiences such as IPV, which could explain the lack of association. Additionally, 46% missing data for the contraceptive use variable, likely due to how the question was framed, could have contributed to the high proportion of missing responses and biased the estimated modern contraceptive use prevalence. The question on ‘current use of family planning methods’ likely led to responses only from women using contraception to prevent pregnancy, excluding those using it for STI prevention or who did not consider family planning. This likely led to a high amount of missing data and biased the estimated contraceptive use prevalence compared to national statistics. Furthermore, grouping all modern contraceptives together may also mask potential associations with specific contraceptive methods, like condom use, which may be more directly influenced by IPV. Taken together, these methodological and contextual factors help explain the higher contraceptive prevalence observed in our study compared to national data and the lack of association between IPV and contraceptive use. Further research is needed to clarify the IPV-contraceptive use relationship in Uganda.

### Strengths and limitations

The findings of this study should be interpreted in light of certain limitations. A significant amount of missing data in the IPV exposure variable could bias the odds ratios. Around 20% of participants reported ‘Not applicable/No partner’ for IPV questions, likely due to not having a partner in the past 12 months. Of this 20% of missing answers, 32.5% came from the age group 15–24 years old. It is plausible that many younger women did not have an intimate partner in the last 12 months.

The assessment of unintended pregnancy may be inaccurate due to the stigma, as some women may hesitate to disclose such information. Studies suggest that women tend to report pregnancies as intended when asked retrospectively after the child is born, even if initially unintended [43,44]. Additionally, 9% of the data on unintended pregnancy was missing, with the highest missing rate (21%) among the youngest age group, 15–24, likely because younger women probably had less opportunities to experience an unintended pregnancy in their lifetimes.

Contraceptive use had 40% missing data, with, 51% of it from the those aged 15–24. We primarily attributed the missing data to the non-response among participants under 18 years of age. This is likely because younger, school-going girls who are not sexually active and/or abstain from sexual activities due to fear of pregnancy and therefore do not use contraceptives. A study by Wondimagegne et al. [45] in neighbouring Ethiopia indicates that lack of contraceptive services in schools and fear of unintended pregnancy among others were the reasons for non-use of contraceptives among adolescent girls. This non-response could introduce some bias, but given their few numbers, our additional analyses to assess the potential effect indicated our findings remain largely consistent. In addition, social desirability could also play a role, as strict social norms in Uganda stigmatize contraceptive use among young girls and women [46].

The strength of this study lies on its large sample size, capturing women, from urban, semi-urban, and rural areas. Around 50% reported psychological IPV, whereas reports of sexual and physical violence were lower, possibly due to stigma and underreporting. It is also possible that the question on psychological violence did not fully capture its nature as we did not use a full standardized module for its assessments. Future studies could improve data accuracy by using full standardized module for assessing psychological violence in Ugandan context.

This study was population-based, involving random selection of participants, and relatively large sample size, both of which enhance the generalizability of the findings. Although the sample size was large, the survey’s response rate was 77% which is slightly below the ideal 80%, and as such, and the finding should be interpreted with caution. Further information about the cohort can be found elsewhere [34]. Additionally, before this study was undertaken, we performed preliminary checks to assess the representativeness and robustness of our data by comparing respondents and non-respondents on available demographic factors, and key variables. These checks revealed no significant differences between the two groups indicating that non-response bias was minimal. However, it is unclear if all registered individuals were invited to participate in the survey, complicating generalization of the results to the broader Ugandan population.

While confounders were adjusted for, residual confounding may still exist. Furthermore, given the cross-sectional design, it is not possible to establish causation or temporality; results should be viewed as associations. It is possible that women were already HIV positive or had unintended pregnancies prior to experiencing IPV. Longitudinal studies are needed to assess the temporality of IPV and its impact on SRHR.

### Public health implications

This research contributes to the growing body of evidence linking IPV and SRHR among women in low-resource settings. Community-based approaches in Uganda targeting social norms – such as the belief that men should dominate and control of women, particularly in sexual relationships – have aimed to reduce IPV [47], with some studies showing a decrease HIV incidence [48]. The findings suggested that IPV is also associated with unintended pregnancies. Further studies are needed to clarify the mechanisms and temporality of these links and their relationship to contraceptive use. However, the findings are valuable to inform the direction of further research in Uganda, to prevent both IPV and poor SRHR.

## Conclusions

This study provides evidence that sexual IPV is associated with unintended pregnancies. The association between IPV and HIV was not statistically significant, and while the findings suggest a possible link between these variables, further research is required to clarify this potential association among women of reproductive age in Wakiso and Hoima districts. The association between IPV and contraceptive use was found not to be statistically significant and remains unclear. Given the cross-sectional design of the study, causal inference cannot be established, and future longitudinal studies are needed to assess causality between IPV and poor SRHR. However, the results may inform future studies aimed at preventing IPV and improving SRHR outcomes in Uganda.
